# Chromone and Flavonoid Alkaloids: Occurrence and Bioactivity

**DOI:** 10.3390/molecules17010191

**Published:** 2011-12-27

**Authors:** Shahriar Khadem, Robin J. Marles

**Affiliations:** Natural Health Products Directorate, Health Products and Food Branch, Health Canada, 2936 Baseline Road, Ottawa, Ontario, K1A 0K9, Canada

**Keywords:** flavonoids, alkaloids, flavonoid alkaloids, chromone alkaloids, natural occurrence

## Abstract

The chromone and flavonoid alkaloids represent an unusual group of structurally diverse secondary metabolites, derived from the convergence of multiple biosynthetic pathways that are widely distributed through the plant and animal kingdoms. Many of them have been discovered through bioassay-guided chemical investigations of traditional medicines, suggesting potential therapeutic significance. Their unique structures and varied pharmacological activities may provide important new leads for the discovery of drugs with novel mechanisms of action. Potential therapeutic indications are as diverse as cancer and viral infections, inflammation and immunomodulation, neurological and psychiatric conditions, and diabetes.

## 1. Introduction

**Figure 1:**
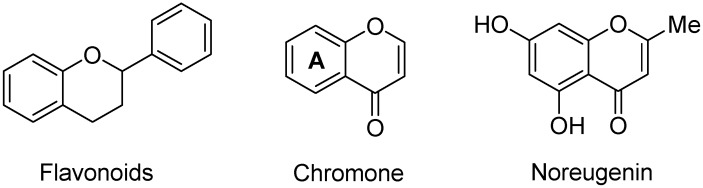
Flavonoid core and chromone structure.

There is absolutely no doubt that natural products have provided key leads for drug discovery [1]. The search for novel natural products with interesting biological properties is an ongoing exercise. Produced by many organisms including humans, alkaloids are a large group of secondary metabolites containing usually basic (in some cases neutral or quaternary) nitrogen derived from an amino acid (or a purine, pyrimidine or other source such as transamination in the case of pseudoalkaloids), in a heterocycle (or aliphatic in the case of protoalkaloids). Most alkaloids are classified chemically according to the nitrogen-containing ring system [2]. However, in terms of chromone and flavonoid alkaloids, this classification is based on the part of the molecule to which the nitrogenous moiety is attached. A structure consisting of a nitrogen system (such as pyridine, piperidine, pyrrolidine) linked to the ‘A’ ring of chromone (Figure 1) is referred to as a chromone alkaloid [3]. This group of compounds can be sub-divided into two types, namely those in which the chromone nucleus exists as noreugenin (5,7-dihydroxy-2-methylchromone) (chromone alkaloids) and those which bear an aryl substituent at *C*-2 (flavonoid alkaloids or ‘flavoalkaloids’) [4]. The flavonoids appearing in the flavoalkaloid structures include flavans, flavons, flavonols, flavanones, flavanonols, and flavan-3-ols (catechins and epicatechins). Chromone and flavonoid alkaloids are of interest not only due to their amphoteric (being both bases and phenols) nature, but because of the pronounced biological activity of some of the natural sources which contain them. In our continuing effort to review less well-known classes of natural products and their bioactivities [5], this article will discuss the natural occurrence and known biological activities of chromone and flavonoid alkaloids. However, those flavoalkaloids with more than two rings (except one tricycle) have not been discussed here for the sake of brevity.

## 2. Results and Discussion

### 2.1. Flavonoid Alkaloids

**Figure 2:**
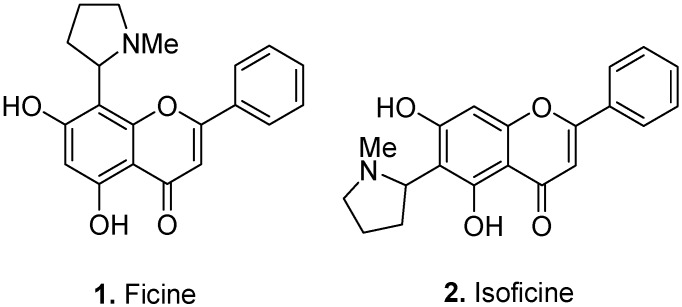
Ficine (**1**) and isoficine (**2**).

The first known flavoalkaloids ficine [5,7-dihydroxy-8-(1-methyl-2-pyrrolidinyl)-2-phenyl-4*H*-1-benzopyran-4-one] (**1**, Figure 2) and isoficine [5,7-dihydroxy-6-(1-methyl-2-pyrrolidinyl)-2-phenyl-4*H*-1-benzopyran-4-one] (**2**) were isolated from the wild fig, *Ficus pantoniana* King, Moraceae [6]. The alleged proteolytic and vermicidal properties of ficine reported in the literature (for example see [2]) are, in fact, related to the enzyme ficin and not the flavoalkaloid ficine.

**Figure 3:**
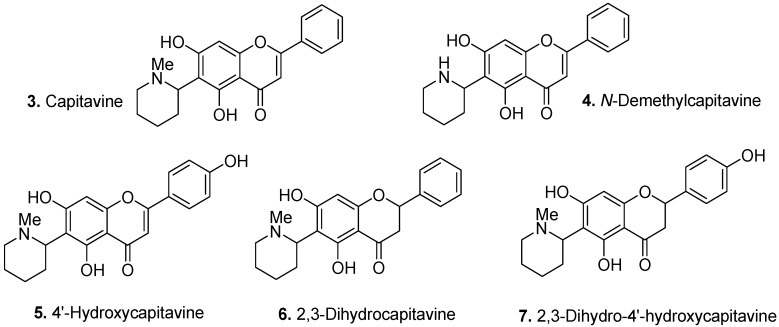
Capitavine (**3**) and capitavine derivatives (**4–7**).

Capitavine [5,7-dihydroxy-6-(1-methylpiperidin-2-yl)flavone] (**3**, Figure 3) is a flavoalkaloid from the seeds of *Buchenavia capitata* Eichler, Combretaceae [7]. Two other capitavine derivatives, namely 4′-hydroxycapitavine [5,7,4′-trihydroxy-6-(*N*-methyl-2″-piperidinyl)flavone] and 2,3-dihydro-4′-hydroxycapitavine [5,7,4′-trihydroxy-6-(*N*-methyl-2″-piperidinyl)flavanone] (**5** and **6**, respectively) were also isolated from the same plant. However, *N*-demethylcapitavine and 2,3-dihydrocapitavine [5,7-dihydroxy-6-(*N*-methyl-2″-piperidinyl)flavanone] (**4** and **7**, respectively) were found only in the fruits of *Buchenavia macrophylla* Eichler.

**Figure 4:**
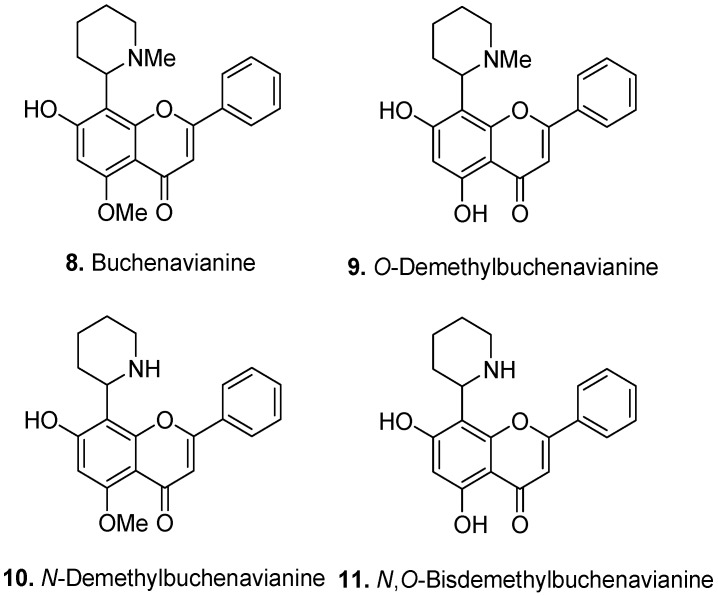
Buchenavianine (**8**) and Buchenavianine derivatives (**9–11**).

Buchenavianine (**8**, Figure 4) is the major alkaloid from the leaves of *B. macrophylla* and is also found in *B. capitata* [7]. *O*-Demethylbuchenavianine (**9**), which is found in both species, showed anti-HIV activity [8]. *N*-Demethylbuchenavianine (**10**) is another alkaloid from the leaves and fruits of *B. macrophylla*. Finally, *N*,*O*-bisdemethylbuchenavianine [5,7-dihydroxy-8-(2-piperidinyl)flavone] (**11**) has been isolated from the fruits of *B. macrophylla* [7]. 

Extensive and ongoing research has been conducted and funded by the U.S. National Center for Complementary and Alternative Medicine and the U.S. National Cancer Institute in support of the discovery of selective anti-HIV and anti-cancer drugs from natural sources. Therefore, the identification of novel molecules and their congeners such as these flavoalkaloids with relevant bioactivities provides important leads for further drug development including structure-activity relationship analysis.

**Figure 5:**
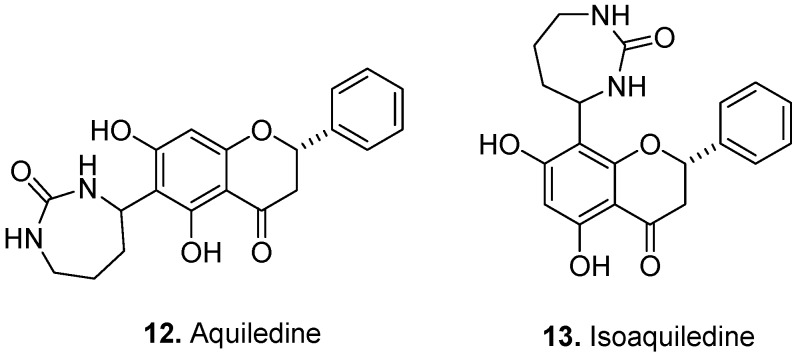
Aquiledine (**12**) and isoaquiledine (**13**).

*Aquilegia ecalcarata* Maxim., Ranunculaceae, Chinese Columbine, is a Chinese medicinal plant used for the treatment of pustulosis, necrotic boils, and other infections [9]. Aquiledine [(2*S*)-6-(1,4-ureylenebutyl)-5,7-dihydroxyflavanone] (**12**, Figure 5) and isoaquiledine [(2*S*)-8-(1,4-ureylenebutyl)-5,7-dihydroxyflavanone] (**13**) were isolated from *A. ecalcarata* [10,11]. Their structures contain a saturated 1,3-diazepin-2-one ring attached to *C*-6 and *C*-8 positions of the flavonone.

**Figure 6:**
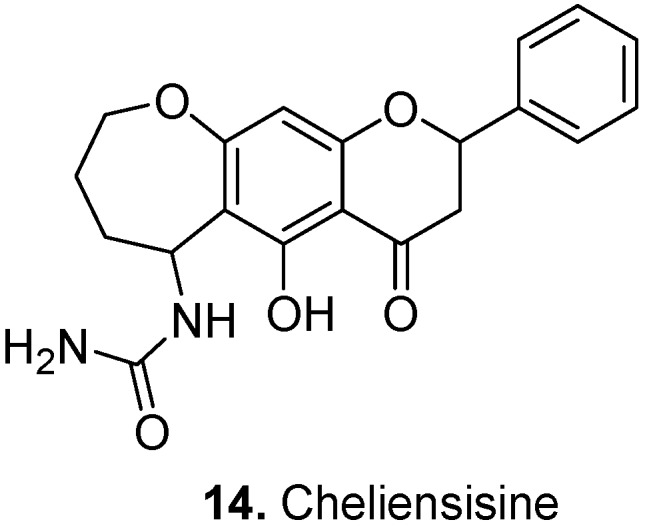
Cheliensisine (**14**).

Cheliensisine (**14**, Figure 6) was isolated from *Goniothalamus cheliensis* Hu, Annonaceae, a plant growing in China. This plant has shown antineoplastic activity [12]. Cheliensisine belongs to the oxepinochromone family of natural products, for which syntheses are being developed due to their unusual structures and potential for drug development (for example, see [13]).

**Figure 7:**
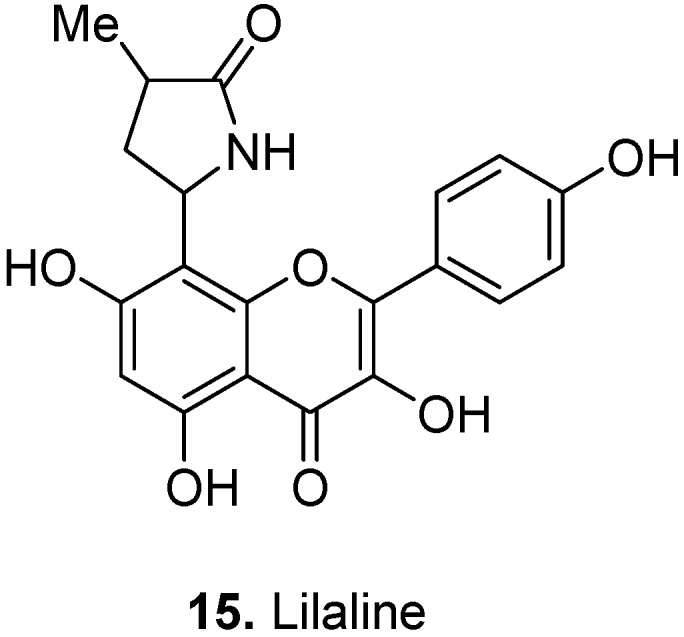
Lilaline (**15**).

*Lilium candidum* L., Liliaceae, Madonna lily, is a plant found in eastern Europe and western Asia. A pyrrolidinoflavonol called lilaline [3,4′,5,7-tetrahydroxy-8-(4-methyl-5-oxo-2-pyrrolidinyl)-flavone] (**15**, Figure 7) was isolated from the aerial parts (flowers) [14].

In traditional medicine, it is believed that the flower of *L. candidum* has anti-inflammatory properties [15]. The relevance of the flavonoid constituents of lily flowers to anti-inflammatory activities such as cyclooxygenase inhibition has been demonstrated (for example, see [16]).

**Figure 8:**
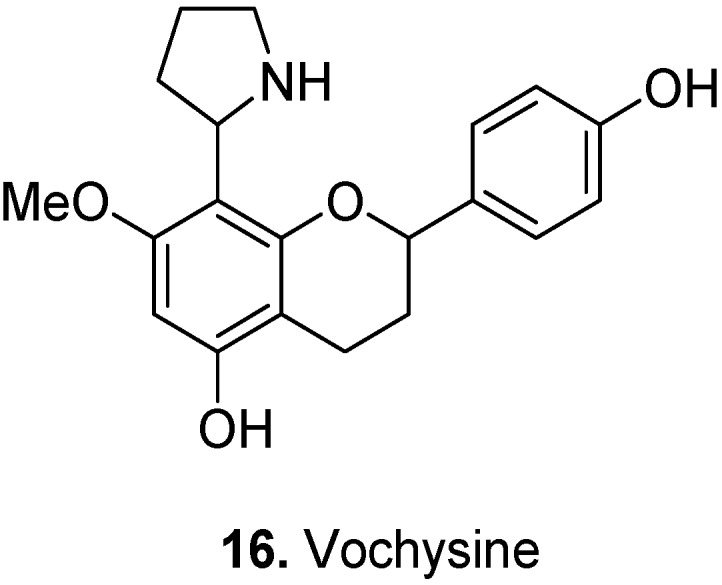
Vochysine (**16**).

The only known pyrrolidinoflavan, vochysine [3,4-dihydro-2-(4-hydroxyphenyl)-7-methoxy-8-(2-pyrrolidinyl)-2*H*-1-benzopyran-5-ol] (**16**, Figure 8) was isolated from the fruits of the Amazonian tree *Vochysia guianensis* Aubl., Vochysiaceae [17].

**Figure 9:**
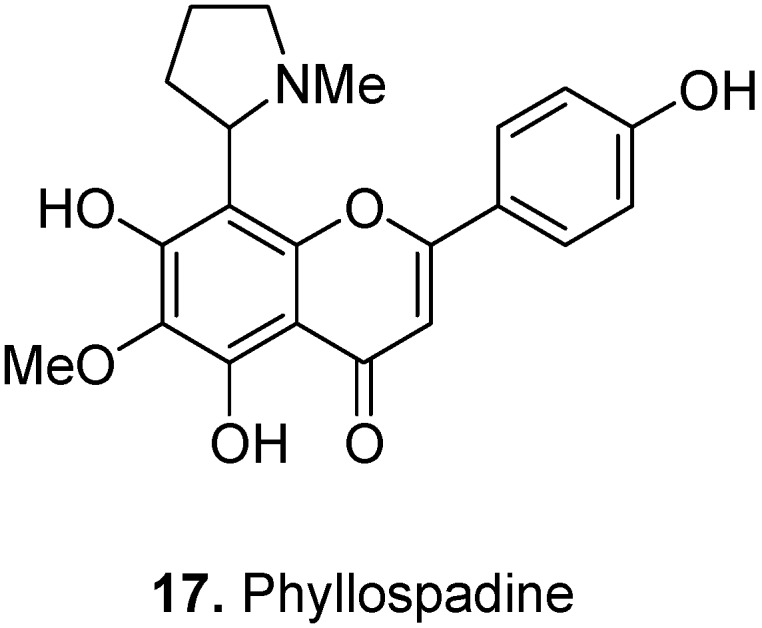
Phyllospadine (**17**).

Sea-grass, *Phyllospadix iwatensis* Makino, Zosteraceae, grows in the coastal region of Japan and Russia. The flavoalkaloid, phyllospadine [4′,5,7-trihydroxy-6-methoxy-8-(1-methyl-2-pyrrolidinyl) flavone] (**17**, Figure 9) was isolated from this plant [18]. Phyllospadine is similar to two other flavoalkaloids, ficine and isoficine in terms of possessing an *N*-methylpyrrolidinyl substitution (Figure 2).

**Figure 10:**
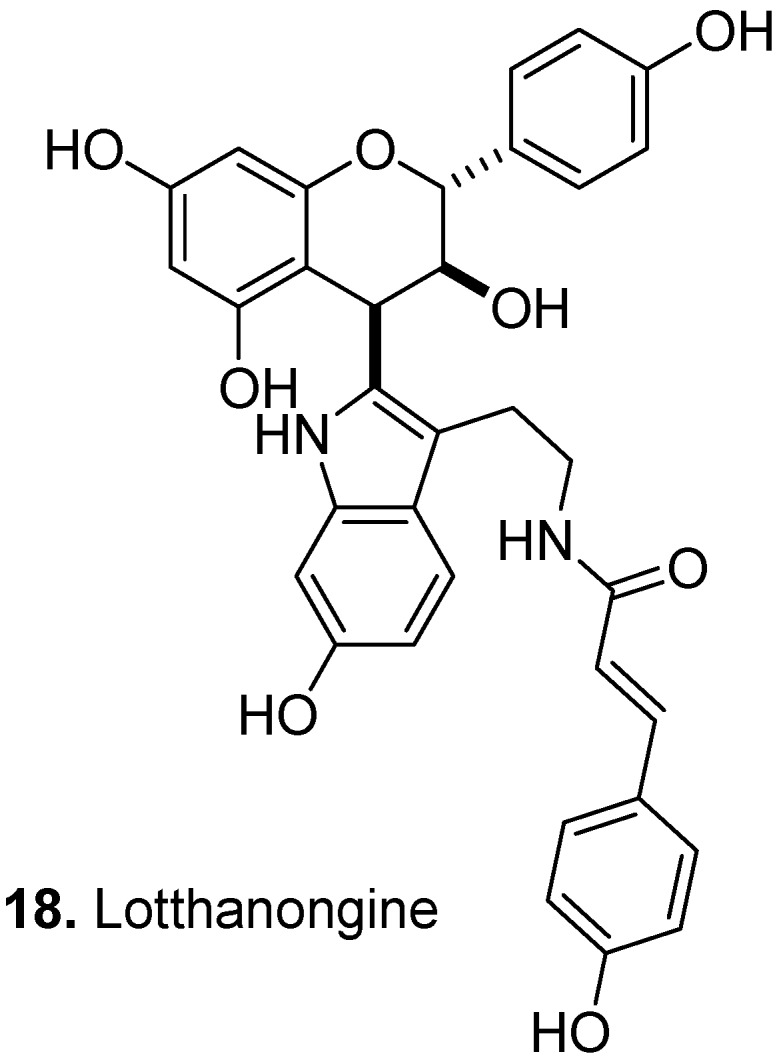
Lotthanongine (**18**).

Lotthanongine [afzelechin-(4β→2″)-N-(p-coumaroyl)-6″-hydroxytryptamine] (**18**, Figure 10) is a flavoalkaloid isolated from the roots of *Trigonostemon reidioides* Craib, (a synonym for *Baliospermum reidioides* Kurz), Euphorbiaceae [19]. In Thai traditional medicine, *T. reidioides* is used as an antidote for poisonous mushrooms, as an expectorant, laxative, and topical antiseptic. Lotthanongine contains an indole moiety attached to the *C*-4 position of an afzelechin molecule. There are many scientific publications on possible mechanisms by which flavonoid derivatives may be hepatoprotective, which would be relevant to a putative mechanism of the traditional antidote for poisonous mushrooms and as a valuable direction for future research on these flavoalkaloids.

**Figure 11:**
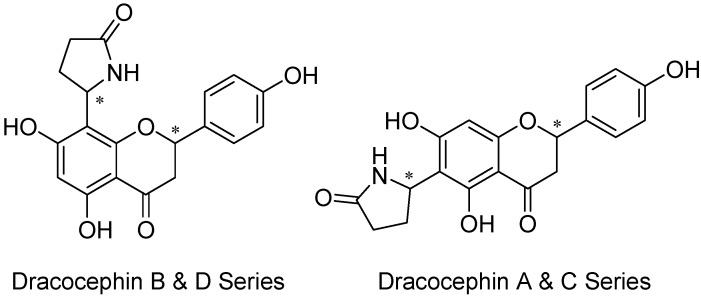
Dracocephins A (**19–22**), B (**23–26**), C (**27–30**), and D (**31–34**).

A widely distributed member of the mint family in western China, *Dracocephalum rupestre* Hance, Lamiaceae, has been used in the treatment of cold, cough, icterohepatitis, laryngalgia, and various other diseases [10]. Sixteen flavoalkaloids called dracocephins [e.g., 4′,5,7-trihydroxy-6-(5-oxo-2-pyrrolidinyl)flavanone and 3,4′,5,7-tetrahydroxy-6-(5-oxo-2-pyrrolidinyl)flavanone] were found in the aerial parts of this plant [18]. Dracocephins A–D (**19–34**, Figure 11) were determined to be mixtures of two diastereoisomeric pairs of enantiomers. Two chiral centers, *C*-2 and *C*″-5, are responsible for this stereoisomerism. Dracocephins are the conjugates of a flavanone (naringenin for A and B or eriodictyol for C and D) with pyrrolidin-2-one (pyrrolidinone). As shown in Figure 11, the pyrrolidinone ring is attached to the *C*-6 position of the flavanone in dracocephins A and C and to the *C*-8 position in dracocephins B and D [21]. As suggested by the traditional uses above, in addition to the extensive body of literature on their anti-inflammatory and immunomodulatory activities, recent research has also shown flavonoid derivatives’ analgesic activity to be another field worthy of further investigation (for example, see [22]).

**Figure 12:**
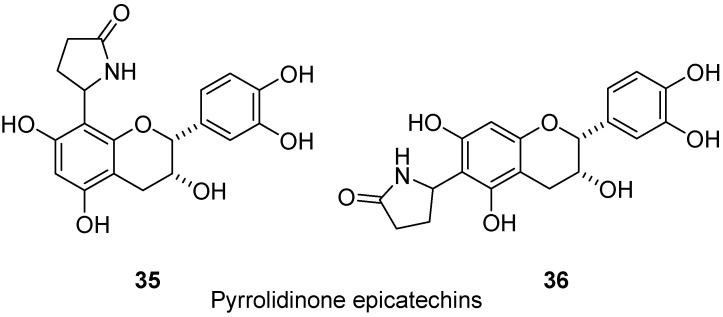
Pyrrolidinone epicatechins (**35** and **36**).

Hyperglycemia affects the pathogenesis of diabetic complications by increasing protein glycation and the build-up of the advanced glycation end products (AGEs) in body tissues [23]. Two pyrrolidinone epicathechins, 6-(2-pyrrolidinone-5-yl)-(−)-epicatechin (**35**, Figure 12) and 8-(2-pyrrolidinone-5-yl)-(−)-epicatechin (**36**), isolated from the roots of the tara vine, *Actinidia arguta* (Siebold & Zucc.) Planch. ex Miq., Actinidiaceae, exhibited significant *in vivo* inhibitory activity against AGEs formation [24]. Even under a physician’s care, the American and Canadian Diabetes Associations report a very high prevalence of potentially life-threatening complications of diabetes including heart disease and stroke (cause of death in up to 80% of diabetics), blindness, kidney disease, nerve damage, erectile dysfunction and depression. For that reason any new leads, such as epicatechin-alkaloid derivatives, for pharmacological interventions to reduce or prevent diabetic complications are extremely important. 

**Figure 13:**
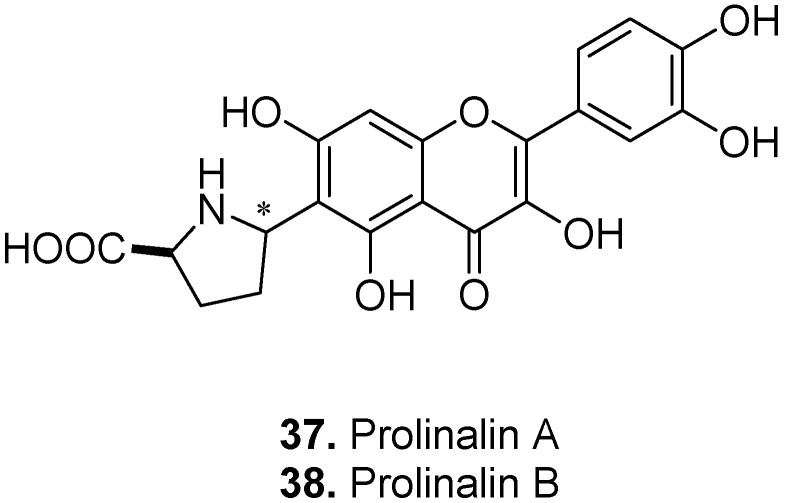
Prolinan A (**37**) and B (**38**).

Prolinalin A [6-*C*-[(2*S*,5*S*)-prolin-5-yl] quercetin] (**37**, Figure 13) and its 5″-epimer prolinalin B [6-*C*-[(2*S*,5*R*)-prolin-5-yl] quercetin] (**38**) are two *C*-prolinylquercetins isolated from the yellow cocoon shell of the silkworm, *Bombyx mori* L., Bombycidae [25]. Prolinalins are the first examples of a flavonoid (flavonol) conjugated with an amino acid (*i.e.*, proline). The fact that these flavoalkaloids are only found in the silkworm and not in its host plant (*i.e.*, mulberry) indicates that prolinalins are produced from dietary quercetin within the insect. This suggests that prolinalins from the diet are modiﬁed by a glucosyltransferase within the silkworm that can transfer a glucose residue to the *C*-5 hydroxy position of quercetin.

**Figure 14:**
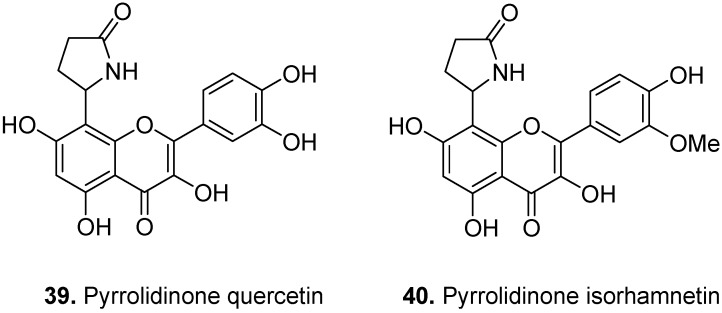
Pyrrolidinone quercetin (**39**) and pyrrolidinone isorhamnetin (**40**).

*Senecio argunensis* Turcz., Asteraceae, is a plant growing in China. It is often used as folk medicine displaying antipyretic effects and detoxicant properties against dysentery [26]. Two pyrrolidinone flavonols namely ‘8-(2″-pyrrolidinone-5″-yl)-quercetin’ (**39**, Figure 14) and ‘8-(2″-pyrrolidinone-5″-yl)-isorhamnetin’ (**40**) were isolated from *S. argunensis* [27].

**Figure 15:**
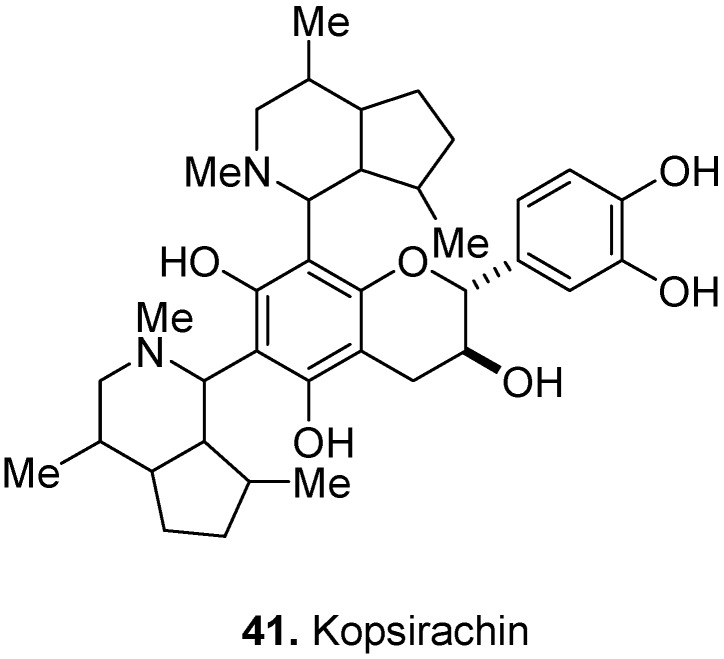
Kopsirachin (**41**).

Kopsirachin [2-(3,4-dihydroxyphenyl)-3,4-dihydro-6,8-bis(octahydro-2,4,7-trimethyl-1*H*-2-pyridin-1-yl)-2*H*-1-benzopyran-3,5,7-triol] (**41**, Figure 15) is a flavoalkaloid isolated from the leaves of *Kopsia dasyrachis* Ridl., Apocynaceae [28]. It consists of two skytanthine moieties linked to the *C*-6 and *C*-8 positions of a catechin molecule.

**Figure 16:**
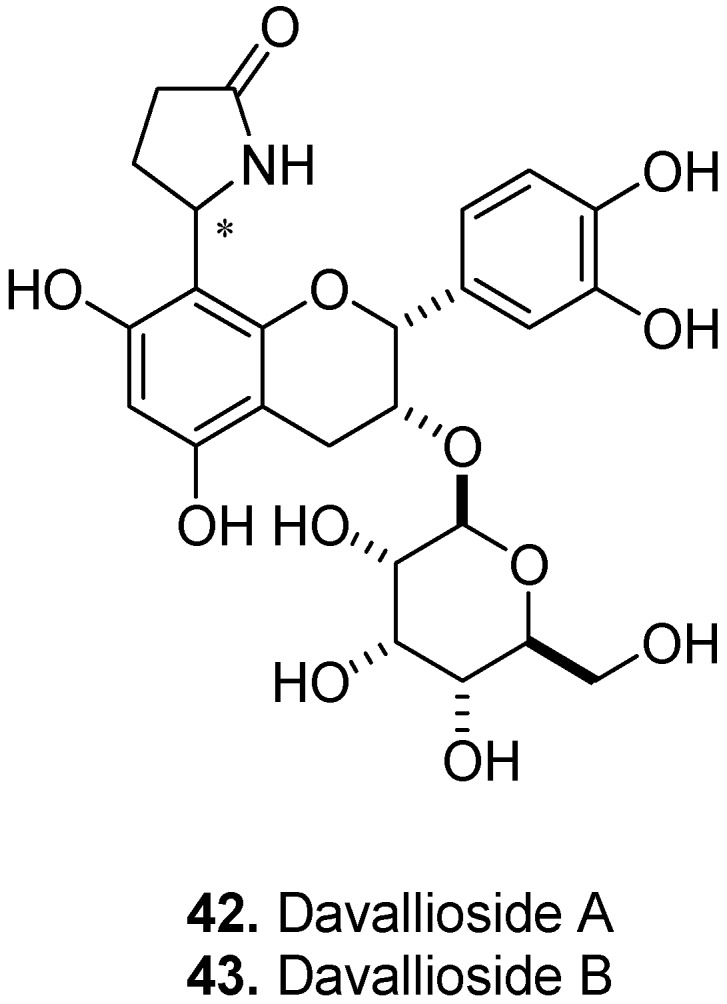
Davallioside A (**42**) and Davallioside B (**43**).

As a folk medicine, the fern, *Davallia mariesii* T. Moore ex Baker, Davalliaceae (also placed in the Dryopteridaceae), is used in Korea to treat common cold, neuralgia, and stomach cancer; in China it is used to treat lumbago, rheumatalgia, toothache and tinnitus [29]. Davallioside A (**42**, Figure 16) and its 1″-epimer Davallioside B (**43**) are constituents of the rhizomes [30].

They are epicatechin glycosides carrying a *γ*-lactam (pyrrolidinone) substituent [8-(2-pyrrolidinone-5-yl)-epicatechin-3-*O*-*β*-D-allopyranoside diastereomers].

**Figure 17:**
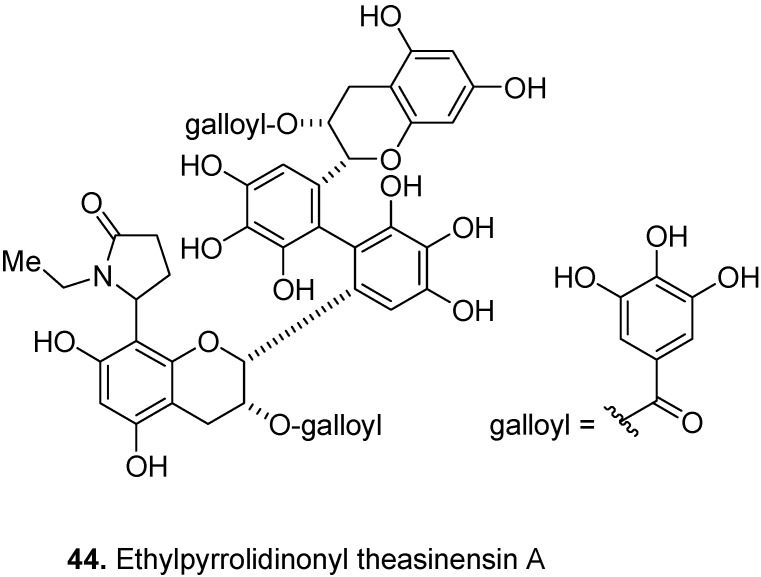
Ethylpyrrolidinonyl theasinensin A (**44**).

Ethylpyrrolidinonyl theasinensin A (8′-ethylpyrrolidinonyltheasinensin A) (**44**, Figure 17) is a dimeric polyphenol having an *N*-ethyl-2-pyrrolidinone moiety. It was isolated from black tea, *Camellia sinensis* (L.) Kuntze, Theaceae [31]. It has been hypothesized that the Strecker aldehydes of tea amino acids, such as L-theanine, may be formed during the drying and enzyme deactivation stages of black tea production. These aldehydes can attack the A-rings of existed catechins, e.g., theasinensin, to form compounds such as **44** [32].

### 2.2. Chromone Alkaloids

**Figure 18:**
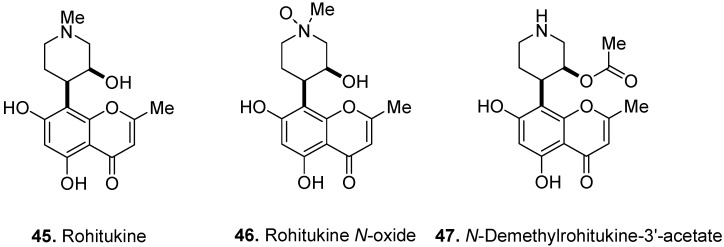
Rohitukine (**45**) and rohitukine derivatives (**46** and **47**).

Rohitukine [5,7-dihydroxy-2-methyl-8-[4-(3-hydroxy-1-methyl)-piperidinyl]-4*H*-1-benzopyran-4-one] (**45**, Figure 18) is a chromone alkaloid from the leaves and stems of *Amoora rohituka* (Roxb.) Wight & Arn. (a synonym of *Aphanamixis polystachya* (Wall.) R. Parker), Meliaceae [33], the stem bark of *Dysoxylum binectariferum* (Roxb.) Hook. F. ex Hiern (a synonym of *D. gotadhora* (Buch.-Ham.) Mabb., Meliaceae [34], the bark of *Schumanniophyton magnificum* Harms (a synonym of *Tetrastigma magnificum* K. Schum.), Rubiaceae [35], and the bark of *Schumanniophyton problematicum* (A. Chev.) Aubrév. (a synonym of *Assidora problematica* A. Chev.), Rubiaceae [36]. Rohitukine showed antiinflammatory and immunomodulatory activities [37]. It also showed moderate cytotoxicity against human HL-60 promyelocytic leukemia and HCT-116 colon cancer cells [38]. Two other chromone alkaloids, rohitukine *N*-oxide (**46**, Figure 18) and *N*-demethylrohitukine-3′-acetate (**47**), were also isolated from the stem bark of *D. binectariferum* [34] and *S. magnificum* [35], respectively. *N*-Demethylrohitukine-3′-acetate is found as a mixture of the 3′α- and 3′*β*-isomers in a 2:1 ratio. According to the U.S. National Cancer Institute, chemotherapy is often given as a combination of drugs. Combinations usually work better than single drugs because different drugs kill cancer cells in different ways. Thus, new leads such as the chromone alkaloids may prove to be valuable complements to the existing armamentarium of antineoplastic agents.

**Figure 19:**
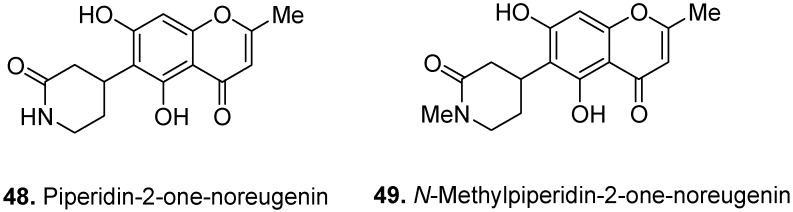
Piperidin-2-one-noreugenin (**48**) and *N*-methylpiperidin-2-one-noreugenin (**49**).

The piperidinone noreugenin [4-(5,7-dihydroxy-2-methyl-4-oxo-4*H*-chromen-6-yl)-piperidin-2-one] (**48**, Figure 19) and its *N*-methyl derivative [4-(5,7-dihydroxy-2-methyl-4-oxo-4*H*-chromen-6-yl)-1-methyl-piperidin-2-one] (**49**) were isolated from the root bark of *S. problematicum*, a west-African plant [39]. In traditional medicine, the alcoholic extract from root of *S. problematicum* is used for treatment of agitated psychotic patients. In fact, the root extract of the plant showed interesting neuropsychopharmacological activities [40]. In addition to the above mentioned compounds, several complex chromone alkaloids, such as schumanniophytine, have also been isolated from *Schumanniophyton* species.

**Figure 20:**
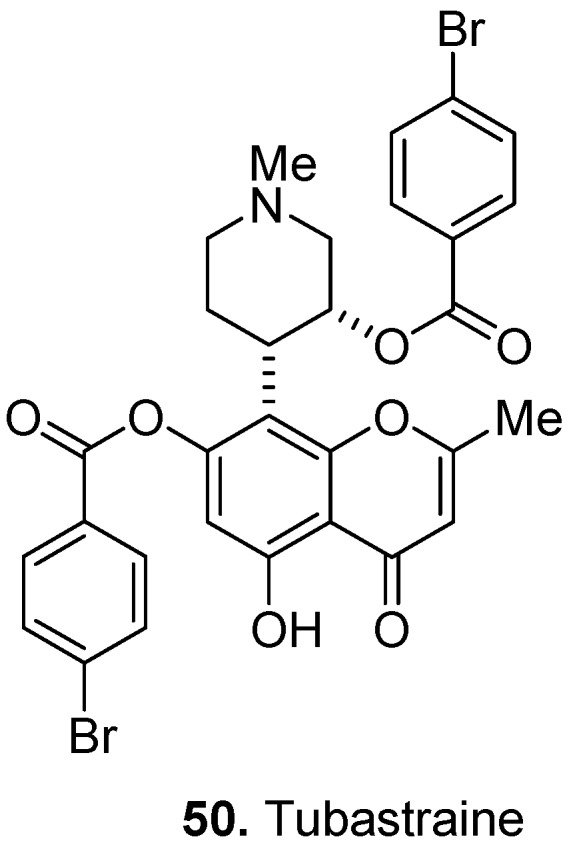
Tubastraine (**50**).

Tubastraine [(3*R*,4*S*)-4-[7-[(4-bromobenzoyl)oxy]-5-hydroxy-2-methyl-4-oxo-4*H*-1-benzopyran-8-yl]-1-methyl-3-piperidinylester, 4-bromobenzoic acid] (**50**, Figure 20) is the first example of a chromone-containing metabolite of a marine invertebrate. This organobromine compound was isolated from the Pacific stony coral *Tubastrea micrantha* Ehrenberg, Dendrophylliidae (black turret coral) [41]. Tubastraine is believed to be responsible for the deterrent response to the crown-of-thorn seastar, *Acanthaster planci* L., Acanthasteridae, the major predator of stony corals. These predator deterrent substances from marine organisms are significant not only from an ecological perspective but also from a therapeutic perspective. The Developmental Therapeutics Program, Division of Cancer Treatment and Diagnosis, U.S. National Cancer Institute, is currently screening natural product materials derived from marine macro-organisms and micro-organisms as potential sources of novel anticancer drugs.

**Figure 21:**
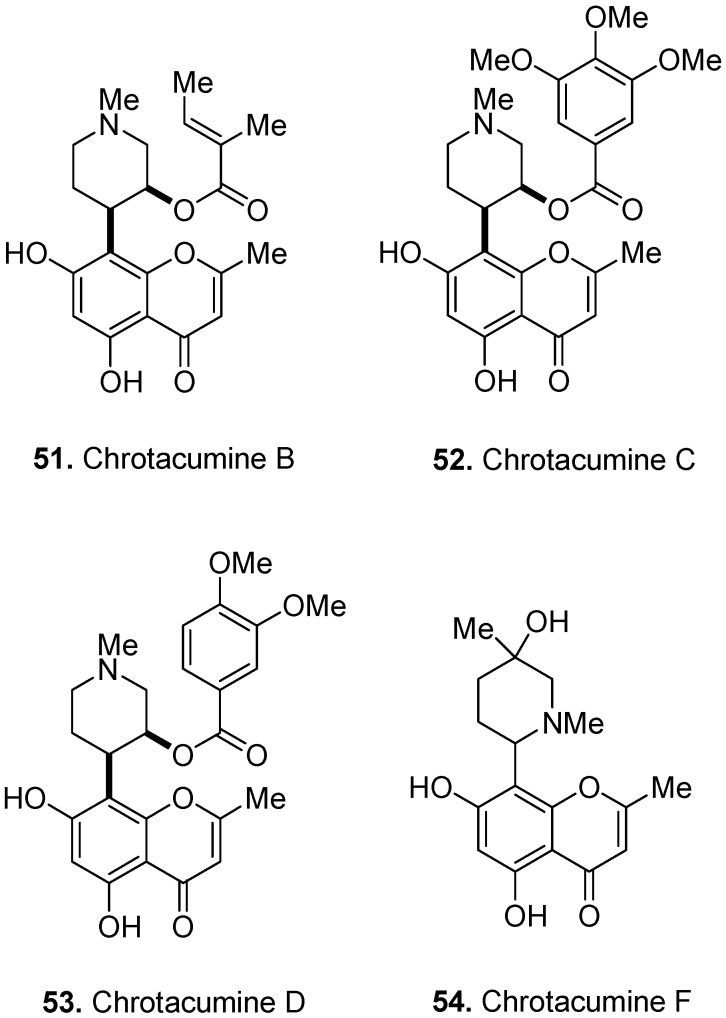
Chrotacumine B (**51**), C (**52**), D (**53**), and F (**54**).

Several chromone alkaloids have been isolated from leaves and bark of *Dysoxylum acutangulum* Miq., Meliaceae [38,42], including chrotacumine B (**51**, Figure 21), C (**52**), D (**53**), and F (**54**). Other chrotacumines, e.g., A and E, are not presented here due to their more complex structures (tetracycles). The chrotacumines shown here share an *N*-methyl piperidine ring attached to a noreugenin molecule. Chrotacumines B, C, and D have additional ester side chains.

**Figure 22:**
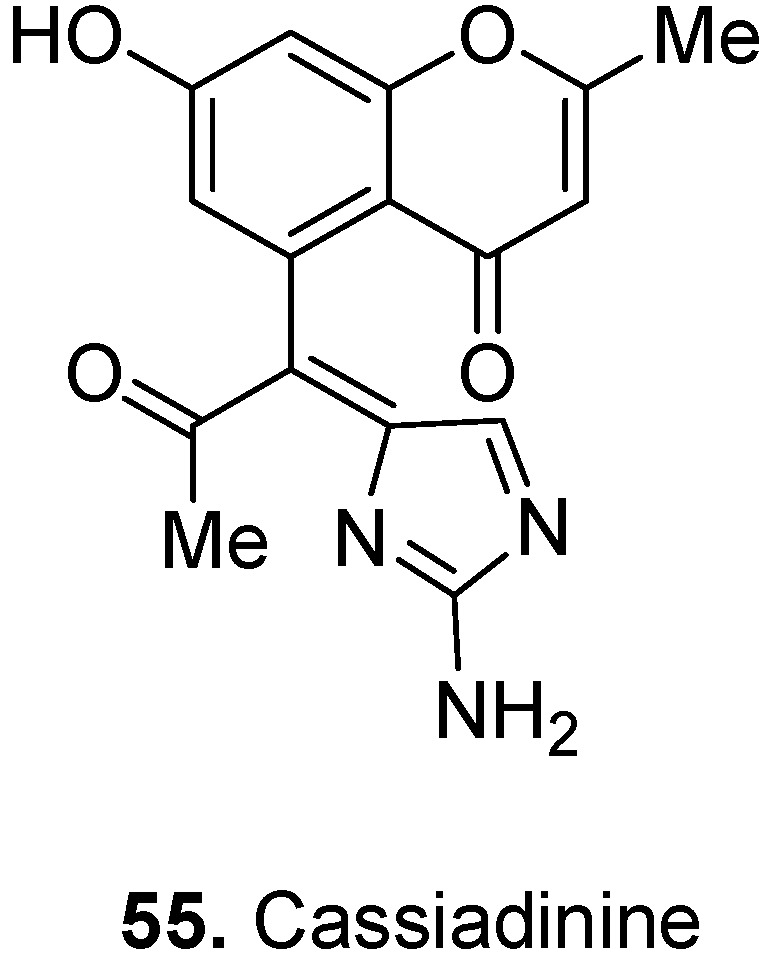
Cassiadinine (**55**).

The flowers of the common Indian tree, Siamese senna, *Cassia siamea* Lam. (a synonym of *Senna siamea* (Lam.) H.S. Irwin & Barneby), Fabaceae, are used to treat insomnia and asthma and their extract has strong antioxidant activity [43]. A chromone alkaloid with unique structural features called cassiadinine [5-[(1*Z*)-1-(2-amino-4*H*-imidazol-4-ylidene)-2-oxopropyl]-7-hydroxy-2-methyl-4*H*-benzopyran-4-one] (**55**, Figure 22) was isolated from the flowers [44]. Cassiadinine contains an acetonylchromone attached to a guanidine moiety. Through their effects to reduce levels of free radicals, antioxidants are intimately involved in the prevention of cellular damage, a common pathway for a variety of diseases, including cancer, and aging. That is another reason why polyphenols such as the flavonoid and chromone alkaloids are potentially valuable drug development leads.

## 3. Conclusions

The chromone and flavonoid alkaloids are of particular interest for several reasons. They are not common but are widely distributed in at least 17 families of plants, including ferns, monocots, and from the most primitive to the most advanced orders of dicots. They have also been reported from two very widely separated types of animals, the insects and the corals, with respect to which evidence suggests endogenous biosynthesis rather than bioaccumulation from a dietary source. This genetic distance between source organisms suggests convergent evolution to similar approaches of chromone and flavonoid alkaloid chemical defenses against herbivores/predators. Such cases of convergent evolution imply a highly successful strategy that confers a survival advantage on the host. They also imply a strong potential for the discovery of further novel natural products with interesting bioactivities from organisms producing chromone and flavonoid alkaloids.

From a biochemical perspective, chromone and flavonoid alkaloids represent a very interesting convergence of multiple biosynthetic pathways, e.g., the shikimic acid pathway to the flavonoids, or the acetate to pentaketide pathway to the chromones, with addition of a nitrogenous moiety coming from L-ornithine to pyrrolidine, L-lysine to piperidine, or from other sources. The result is a group of secondary metabolites with diverse and unique structures.

With respect to bioactivities, many of these flavonoid and chromone alkaloids have been discovered through bioassay-guided chemical investigations of traditional medicines, suggesting that they have significant potential for drug discovery. As could be anticipated, the literature reports ecologically important activities of these substances against predators and pathogens, which often correlate with cytotoxic and antiviral activities. From a drug development perspective, the key challenge will be to determine, and attempt through derivatization to enhance the selectivity of these compounds, for example as targeted antineoplastic rather than generally cytotoxic agents, since natural selection tends to favour a broad spectrum of toxicity to predators and pathogens.

Since many alkaloids exhibit toxic effects through mimicking structurally and biosynthetically related neurotransmitters, perhaps the reported potential neuropsychiatric indications for these compounds could also have been predicted. However, other therapeutically important activities that have been discovered for these flavonoid and chromone alkaloids include potential applications against diabetes, inflammation, and immunological disorders.

**Figure 23:**
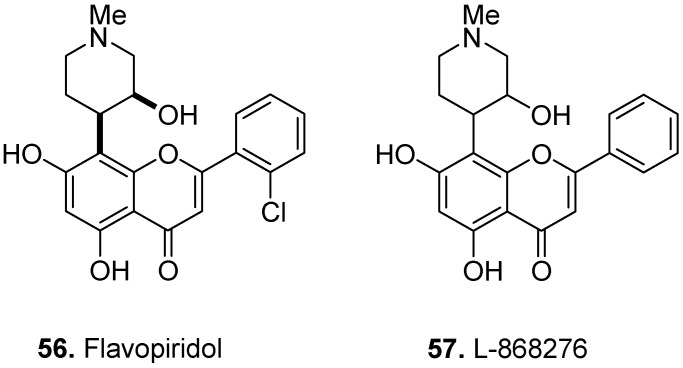
Bioactive semi-synthetic flavoalkaloids: Flavopiridol (**56**) and L-868276 (**57**).

Despite the tremendous success of certain naturally occurring substances as drugs, their limited accessibility and sometimes challenging synthesis often make them only leads and not candidate drugs for scientists and pharmaceutical companies in the quest for new therapeutic hits. Biochemists still need preferably small molecules to demonstrate some degree of perturbation effects on biological processes. One of the most reliable sources of guidance is Mother Nature, through synthesis of natural product-inspired totally synthetic or semi-synthetic compounds [45]. Not surprisingly, some of the flavoalkaloid semi-synthetic compounds have shown interesting bioactivities (Figure 23). For example, flavopiridol (also known as alvocidib) (**56**) is a semi-synthetic flavoalkaloid which inhibited cyclin-dependent kinases CDK1 and CDK2 by alteration of tyrosine phosphorylation of CDK1/CDK2 and competitive inhibition with ATP [46]. As a CDK inhibitor, flavopiridol is a promising agent that induces p53-independent apoptosis in Chronic Lymphocytic Leukemia (CLL) [47]. L-868276 (**57**, Figure 23) is another semi-synthetic compound derived from rohitukine with specific and potent inhibitory effects on CDK2 [48].

In conclusion, the evidence from the literature suggests that these chromone and flavonoid alkaloids have significant bioactivities that likely help their host organisms survive predation and disease. These bioactive compounds may be the basis for the host organisms’ uses in traditional medicines around the world. They have unique and diverse structures that will provide new leads for the discovery of drugs sufficiently different from previous discoveries in the alkaloid world to have perhaps different mechanisms of action and potentially to be effective in some health conditions where there is established multidrug resistance. Furthermore, the spectrum of bioactivities extends beyond those easily anticipated for alkaloids, increasing the potential for the discovery of new therapeutic agents from these unusual natural products. 
